# Occurrence of Guillain-Barré Syndrome in the Early Post-operative Period After an Urgent Liver Transplant

**DOI:** 10.7759/cureus.63304

**Published:** 2024-06-27

**Authors:** Ariel Jasqui-Bucay, Carlos Moctezuma-Velazquez, Geronimo Pacheco-Aispuro, Mario Vilatobá-Chapa, Jonathan Aguirre-Valadez

**Affiliations:** 1 Internal Medicine, Hospital Angeles Lomas, Mexico City, MEX; 2 Medicine, Division of Gastroenterology - Liver Unit, Department of Medicine, University of Alberta, Edmonton, CAN; 3 Neurology, Hospital Angeles Pedregal, Mexico City, MEX; 4 Transplant Department, Instituto Nacional de Ciencias Médicas y Nutrición Salvador Zubirán, Mexico City, MEX; 5 Hepatology, Hospital Angeles Pedregal, Mexico City, MEX

**Keywords:** transplant complication, drug-induced acute liver failure, guillain-barré syndrome, orthotropic liver transplant, drug-induced liver injury (dili)

## Abstract

Solid organ transplant recipients are prone to developing a wide range of complications associated with the procedure itself, as well as with immunosuppressants. Guillain-Barré syndrome, which is part of the spectrum of inflammatory neuropathies, is not expected to occur early after organ transplant when immunosuppression is at its highest point. We describe the clinical case of a patient who underwent an urgent liver transplant due to acute liver failure secondary to drug-induced liver injury and developed Guillain-Barré syndrome early after the transplant.

## Introduction

Neurologic complications are relatively frequent in transplant patients and have been reported in 15-30% of liver transplant (LT) recipients [1-2]. The most frequent complications are the ones targeting the central nervous system, such as seizures and encephalopathy, whereas complications in the peripheral nervous system are rarely seen [1]. Guillain-Barré syndrome (GBS), which is an inflammatory disease of the peripheral nerves and nerve roots and is the most common cause of acute flaccid paralysis, has an annual global incidence of approximately 1-2 per 100,000 person-years [3], and is rarely described in recipients of solid organ transplants [4].

## Case presentation

We present the case of a previously healthy 62-year-old male with no underlying risk factors for chronic liver disease (i.e., no alcohol ingestion and no features of metabolic syndrome) who developed jaundice followed one week later by altered level of consciousness after a 20-day course of nitrofurantoin that had been prescribed for prostatitis. Extensive work-up for metabolic, autoimmune, and infectious liver diseases, including hepatitis E virus infection, was non-contributory, and thus the diagnosis of acute liver failure due to drug-induced liver injury (DILI) was made. The patient was started on N-acetylcysteine and continuous renal replacement therapy and received three sessions of high-volume plasma exchange, but he continued to worsen and eventually met both King’s College and Clichy-Villejuif criteria for LT. He underwent an urgent orthotopic LT from a neurologically deceased donor with an identical blood group.

**Figure 1:**
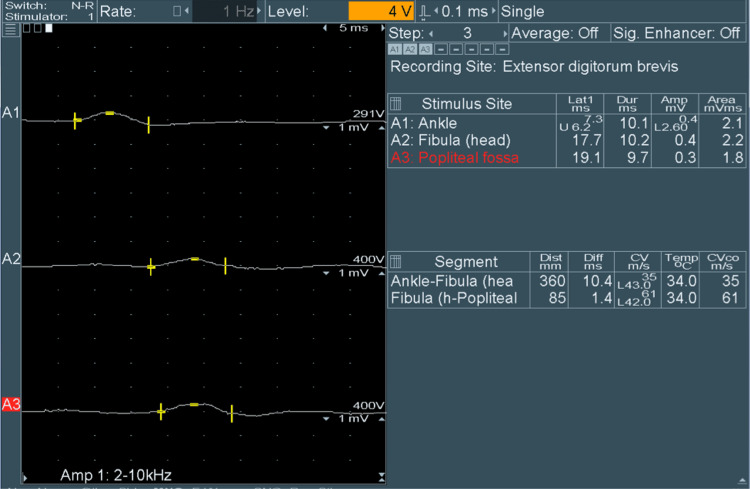
Nerve conduction graph The graph demonstrates the motor nerve conduction responses of the peroneal nerve, with recordings taken from the extensor digitorum brevis muscle and electrical stimulation at the level of the ankle, fibular head, and popliteal fossa on both sides. The compound motor action potentials (CMAPs) show prolonged latencies and decreased amplitudes at each stimulation site on both sides, predominantly on the right, with reduced conduction velocity in the ankle and fibular head segments bilaterally, also predominantly on the right.

The evaluation of the explant showed extensive collapse and necrosis and did not reveal any specific etiology given the scarce parenchyma that was left. Induction immunosuppression was given with steroids and basiliximab, and he was started on mycophenolate mofetil and a prednisone taper in the immediate postoperative period, whereas tacrolimus was delayed and started on the fourth day. His initial course was unremarkable and his neurologic status was back to normal, but after the first 72 hours, he developed marked weakness, predominantly distal, which progressed to flaccid quadriparesis with areflexia. Magnetic resonance of the brain showed no abnormalities. A lumbar puncture was performed and cerebrospinal fluid analysis revealed normal cellularity with elevated proteins, albuminocytological dissociation, and negative infectious workup (i.e., polymerase chain reaction for E. coli, enterovirus, herpes simplex virus, varicella-zoster virus, cytomegalovirus, and Neisseria meningitidis); nucleic acid testing for cytomegalovirus was also negative in blood. Nerve conduction studies (NCS) and electromyography (EMG) revealed polyneuropathy with a predominance of motor axonal and conduction blockages, the latter being related to demyelination, with acute denervation without reinnervation, as shown in Figure 1.

With this, the diagnosis of GBS was made, and he received IVIg for five consecutive days, at a dose of 0.4 mg per kilogram every 24 hours for five days. The total total dose was 2 mg per kilogram. He also developed dysphagia for both solids and liquids. Therefore, due to the inability to eat orally, parenteral nutrition was used, and subsequently feeding via a nasojejunal tube was initiated; later, a percutaneous endoscopic gastrostomy was placed. Over the following days, his course stabilized and with the help of an intense neurological rehabilitation program, he started to improve and was discharged after a two-month hospital stay. He was discharged with the gastrostomy and it was removed a week later after an evaluation to rule out dysphagia was successfully completed. Currently, one year after his LT, he is fully neurologically recovered except for a mild neurosensorial hearing loss.

## Discussion

GBS is an acute onset inflammatory demyelinating polyneuropathy thought to be caused by an aberrant immune response to infections that results in damage to the peripheral nerves, although the pathogenesis is not fully understood [5]. Due to its inflammatory nature, it is not expected in immunocompromised patients, including solid organ transplant recipients, and as a result, there are only isolated case reports of GBS in heart [6], liver [7-9], and kidney transplant recipients [10]. Cytomegalovirus infection is the most commonly reported trigger for GBS in the post‐renal transplant setting [4,10], but vaccines have also been implicated [11-12], as well as calcineurin inhibitors [13], episodes of rejection, and Campylobacter and other infections [7,14]. In the case of our patient, we could not identify a precipitant; Cytomegalovirus and hepatotropic viruses, including hepatitis E virus [15], were ruled out, and tacrolimus was started only after the patient had already developed the initial symptoms of polyneuropathy. He had been vaccinated against influenza and severe acute respiratory syndrome coronavirus 2 (SARS‑CoV‑2), but several months before the LT, making it unlikely to be the cause. It has also been proposed that GBS in transplant patients may be the result of an immune reconstitution inflammatory syndrome [8], but in the case of our patient, who developed the initial manifestations in the early post-LT period, when immunosuppression is at its maximum, and did not receive treatment for an infection, this is unlikely.

The differential diagnosis in this case also included nitrofurantoin-associated neuropathy. Crucial factors, including the onset and progression of symptoms, the symmetry and distribution of muscle weakness, cerebrospinal fluid findings, the findings of the NCS, and the response to treatment, were instrumental in distinguishing between the two conditions. While NCS and EMG are expected to reveal similar changes to those observed in Guillain-Barre syndrome, they do so without conduction blockage. The specific incidence of conduction blockages in polyneuropathy associated with nitrofurantoin use is not well-recorded, given the considerable variability in symptom presentation. In contrast, in this case, it was more in line with GBS, with demyelinating and axonal characteristics [16]. In addition, GBS is consistently treated with IVIg or plasma exchange, resulting in positive outcomes, like in our patient´s case. On the other hand, these treatment methods are not expected to be as effective for drug-induced neuropathy [17].

## Conclusions

Overall, even if the pre-test probability of GBS in solid organ recipients may be lower than that of the general population, it is important to keep it within the differentials of patients with new onset and progressive weakness, as appropriate therapy can change the natural history of this disease. This particular case illustrates how important it is to differentiate between GBS and drug-induced neuropathy, as both conditions can present with similar symptoms. When GBS is diagnosed in transplant recipients, in addition to the most common triggers, it is important to rule out cytomegalovirus infection and calcineurin inhibitor toxicity, as both are factors related to the onset of the disease in these patients. 

This case report reminds clinicians to remain vigilant in their diagnostic approach when encountering complex cases with overlapping symptoms, especially in immunocompromised patients, including those who have undergone solid organ transplantation.
